# Primary School of Medicine

**Published:** 1858-10

**Authors:** 


### PRIMARY SCHOOL OF MEDICINE.

We find upon our table Dr. E. F. Knott's circular, in which he proposes to establish a Primary School of Medicine in Griffin, Ga. The move is a good one. We have long since been impressed with the necessity of a more systematic course of study than is usually pursued by young men commencing the study of medicine.

Physicians with an extensive practice, and more particularly a laborious country practice, have but little time or disposition to devote to those who have selected these offices as a point to commence their course of study. The result is, the student becomes careless and inattentive, or if studious, reads without much benefit; so thus, at the expiration of eight or ten months, he leaves for college not further advanced than he would have been in half that time, with the proper attention of his preceptor. Daily instructions and examinations are more important to those just commencing, than at any period during the entire course of study. Had we more Primary Schools, or more systematic office instruction we would have better physicians.

Dr. Knott is having erected an appropriate building, which will be completed by the first of November, at which time he proposes commencing his course of instruction.

